# Hepcidin Promoted Ferroptosis through Iron Metabolism which Is Associated with DMT1 Signaling Activation in Early Brain Injury following Subarachnoid Hemorrhage

**DOI:** 10.1155/2021/9800794

**Published:** 2021-12-27

**Authors:** Hongxia Zhang, Robert Ostrowski, Dengzhi Jiang, Qing Zhao, Yidan Liang, Xudong Che, Jun Zhao, Xiang Xiang, Wang Qin, Zhaohui He

**Affiliations:** ^1^Department of Neurosurgery, the 1st Affiliated Hospital of Chongqing Medical University, Chong Qing, China; ^2^Department of Experimental and Clinical Neuropathology, Mossakowski Medical Research Centre Polish Academy of Sciences, Warsaw, Poland

## Abstract

Iron metabolism disturbances play an important role in early brain injury (EBI) after subarachnoid hemorrhage (SAH), and hepcidin largely influences iron metabolism. Importantly, iron metabolism may be associated with ferroptosis, recently a nonapoptotic iron-dependent form of cell death that may have a great impact on brain injury after SAH. We investigated hepcidin on iron metabolism and ferroptosis involving divalent metal transporter 1 (DMT1), and ferroportin-1 (FPN1) in a rat model of SAH. Male Sprague-Dawley rats were subjected to the endovascular perforation to induce SAH, and treated with heparin (inhibitor of hepcidin), or oncostatin M (OSM, inducer of hepcidin), or ebselen (inhibitor of DMT1) by intracerebroventricular injections. Hepcidin, DMT1, FPN1 and glutathione peroxidase 4 (GPX4), were detected by western blot and immunofluorescence. Iron metabolism was detected through Perl's iron staining and iron content assay. Ferroptosis, the ROS production, lipid peroxidation (LPO) was evaluated by monitoring methane dicarboxylic aldehyde (MDA), glutathione (GSH), glutathione peroxidase 4 (GPX4) activity, and transmission electron microscopy. Neurological deficit scores, Evans blue staining and brain water content were also determined to detect EBI 72 h after SAH. Our results showed that inhibition of DMT1 by ebselen could suppress iron accumulation and lipid peroxidation, and thereby alleviate ferroptosis and EBI in SAH rats. Heparin downregulated the expression of hepcidin and DMT1, increased FPN1, and exerted protective effects that were equivalent to those of ebselen on ferroptosis and EBI. In addition, OSM increased the expression of hepcidin and DMT1, decreased FPN1, and aggravated ferroptosis and EBI, while the effect on ferroptosis was reversed by ebselen. Therefore, the study revealed that hepcidin could regulate iron metabolism and contribute to ferroptosis via DMT1 signaling activation in rats with EBI after SAH.

## 1. Introduction

Subarachnoid hemorrhage (SAH) is a devastating condition. Increasing evidence indicates that early brain injury (EBI), a recently proposed concept referring to a direct damage to the whole brain within 72 h after SAH, is the most crucial etiological factor of poor clinical prognosis amongst SAH cases [1, 2]. However, the mechanism of EBI in SAH is still poorly understood. Our previous research focused on the iron metabolism in EBI after SAH, it reported that iron metabolism may induce ferroptosis, a new form of cell death. Ferroptosis mainly caused by iron overload and the accumulation of lipid peroxide [3]. It has reported ferroptosis occurs after SAH, and reduction of lipid peroxidation could alleviate ferroptosis in early brain injury after SAH [4]. Whether the disorder of iron metabolism might lead to ferroptosis after SAH has not reported, and the pathway also not. Studies revealed that hepcidin had a potent influence on iron metabolism [5], moreover, our group has found that the expression hepcidin increased in EBI after SAH [6].

**Figure 1:**
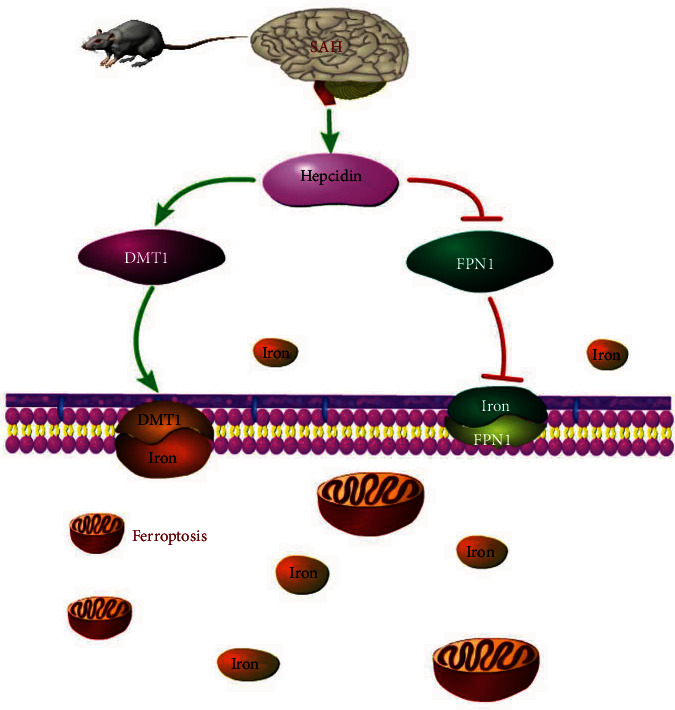
hepcidin might have an effect on iron metabolism and ferroptosis, via DMT1 activation, in EBI after SAH.

Noteworthy, a 2012 study by Dixon et al. indicated that ferroptosis, a nonapoptotic form of cell death, is dependent on intracellular iron-induced accumulation of lipid peroxidation (LPO) products due to reactive oxygen species (ROS), a decreased glutathione (GSH) content, and is manifested by the reduced size of mitochondria [3]. Emerging evidence suggests that iron accumulation and lipid peroxidation, accompanied by reductions in GSH and glutathione peroxidase 4 (GPX4), the main regulator of ferroptosis, which levels can be found in various neurological diseases [7–10]. What's more, the cellular iron-regulatory system is complex and involves various proteins that collectively regulate import, neutralization, storage and export of iron, mainly denoted by the transferrin receptor 1, divalent metal transporter 1 (DMT1), ferritin and ferroportin-1 [11]. Among them, DMT1 protein facilitates iron uptake at the apical cell membrane, and transports iron across the endosomal membrane in almost all cell types that take up iron via transferrin/transferrin receptor 1 pathway [12]. While, ferroportin-1 (FPN1) is one of the extracellular iron transporters, which exports iron across the basolateral membrane. Previous studies indicated that hepcidin, an iron regulatory hormone, could promote iron accumulation through inhibiting FPN1 and ceruloplasmin (CP) and activation DMT1 in the rat cerebral cortex and hippocampus [13]. What's more, ferroptosis was induced by iron deposition, studies found the contrary expression of DMT1 and GPX4 at the same time. Zhang et al. found that lacking Pkd1 exhibit extensive metabolic abnormalities, which include increased expression of iron importers DMT1, which in turn result in high iron levels, low GSH and GPX4 activity, increased lipid peroxidation, and propensity to ferroptosis in autosomal dominant polycystic kidney disease mouse models [14] Zeng et al. showed that a high expression DMT1, at the same time, the low expression of ferroptosis key factor GPX4 was detected in studying the benefits of iron chelators in the treatment of Parkinson's disease [15]. Therefore, we hypothesized that hepcidin could regulate iron metabolism via DMT1 signaling activation and FPN1 inhibition, which promote iron accumulation and induce ferroptosis in EBI after SAH (Figure 1).

Our group have verified that the role of hepcidin might induce apoptosis after EBI in SAH [6], in this study, we further studied the hypothesis of the hepcidin and its pathway on iron metabolism in ferroptosis in SAH. To test the hypothesis, we initially investigated the expression of hepcidin, DMT1, FPN1, and GPX4 in EBI after SAH. We subsequently administered an inhibitor of DMT1, ebselen (also called PZ51), ebselen was shown to significantly decrease iron concentrations [16]. Ebselen treatment is associated with reduced tissue iron in a model of iron overload [17], suggesting its potential inhibition of iron uptake. What's more, it has been demonstrated that ebselen is an inhibitor of DMT1 transport [18, 19]. Ebselen in vivo and investigated the iron metabolism associated with iron content and Perl's iron staining. Moreover, heparin, an inhibitor of hepcidin, was used to investigate the functions of hepcidin in iron metabolism and ferroptosis in EBI induced by SAH. Heparin is a glycosaminoglycan analog to HSPGs, studies showed that heparin is a strong inhibitor of hepcidin expression in hepatic cell lines, in mice, and possibly also in patients with high hepcidin levels, lines also reported evidence that heparin has a strong anti-hepcidin activity in vitro and in vivo [20]. What's more, studies showed that unfractionated heparin strongly inhibits hepcidin expression in HepG2 cells and the unfractionated heparin (UFH, 12-15 k Da) had an effect approximately 10-fold more potent than LMWH (4.5 k Da). To avoid its anticoagulant activity, the glycol-split non-anticoagulant heparin was introduced into research (heparin is abbreviated form of glycol-split heparin in the following text) [21]. Oncostatin M (OSM), an inducer of hepcidin, OSM is an inflammatory cytokine member of the IL-6-related cytokine family, OSM expression was demonstrated in T-cells, monocytes, neutrophils, and also in the testis, brain, and kidne1 [22]. A possible physiologically relevant role for OSM induced hepcidin gene expression could be during fetal liver development, OSM might be more important for hepcidin induction in physio-pathological conditions than IL-6 and that OSM may play an important role in iron dysregulation observed in inflammatory condition [23, 24]. OSM was introduced to investigate the functions of hepcidin in iron metabolism and ferroptosis in EBI induced by SAH. Finally, the mechanism involving DMT1 was also investigated using both ebselen and OSM interventions. Ultimately, our findings reveal that hepcidin and its related signaling pathways after SAH may provide a molecular target for clinical treatment.

## 2. Materials and Methods

### 2.1. Animals and Grouping

Male Sprague-Dawley (SD) rats were introduced into research, for the present SAH model a total of 383 rats, weighing 250–300 g, were purchased from the Animal Center of Chongqing Medical University. The rats had free access to water and food, and were housed in plastic cages with a 12 h light/dark cycle, which was in accordance with the National Institute of Health Guide for the Care and Use of Laboratory Animals (NIH Publications No. 80-23) revised 1996. Before surgery, the rats were fasted for 12 h and deprived of water for 8 h. The animal care and all experimental protocols adhered to the guidelines of and were approved by the Institutional Medical Experimental Animal Care Committee of Chongqing Medical University.

The adult male SD rats assigned to SAH model procedures were randomly divided into several groups. The rats assigned to SAH model procedures were randomly divided into the groups, first to determine the expression of hepcidin, DMT1, FPN1, and GPX4, the main regulator of ferroptosis, and to subsequently select the most suitable timing for drug injections. Second, adult male SD rats were randomly divided into the groups to determine the significant preoperative doses of ebselen, heparin and OSM in terms of their effects on hepcidin, DMT1, FPN1, and GPX4 for further study. Lastly, male SD rats were randomly divided into the groups to determine the effects of hepcidin and DMT1 on iron metabolism, ferroptosis, and EBI, by using heparin, ebselen and OSM as the experimental interventions.

### 2.2. Rat Model of SAH

The endovascular perforation model is considered be in similar with the pathophysiological changes observed with a clinical ruptured intracranial aneurysm, which is the most suitable model of SAH [25]. Takashi Sugawara et.al outlined a simple and objective novel SAH grading system by examining endovascular perforation of subarachnoid blood clots in the basal castle and evaluating its correlation with neurological status [26]. They found that the new SAH severity scale was linearly associated with neurological dysfunction, which is a more intuitive and effective evaluation of the SAH model. SAH animal models were established through endovascular perforation as previously described [27]. The rats were anesthetized by intraperitoneal injection of pentobarbital sodium (50 mg/kg), and a sharpened 4-0 monofilament nylon suture was inserted into the right internal artery from the stump of the external carotid artery to puncture the bifurcation of the anterior and middle cerebral arteries. The sham-operated group underwent the same procedure as the SAH group, but without puncturing the vessel wall. As mentioned earlier, at the time of euthanasia, the severity of SAH was assessed by investigators who were unaware of the group ID, and rats with scores <9 were excluded from the study.

### 2.3. Treatments

#### 2.3.1. Treatment 1

**Figure 2:**
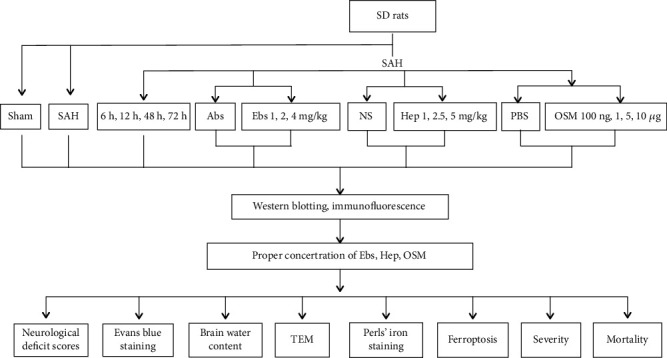
The proper concentration of ebselen, heparin and OSM were chosen to evaluate the ferroptosis and EBI after SAH.

Western blot was performed to determine the expression profiles of hepcidin, DMT1, FPN1, and GPX4 at 6, 12, 24, 48, and 72 h after SAH (*n* = 6, each group, Figure 2).

#### 2.3.2. Treatment 2

Ebselen (DMT1 inhibitor) at doses of 1, 2, and 4 mg/kg was injected intracerebroventricularly 24 h before SAH constructed, the vehicle groups received 10% absolute alcohol (Abs) injection (*n* = 6, each group, Figure 2). Heparin (hepcidin inhibitor) at doses of 1, 2.5, and 5 mg/kg was injected intracerebroventricularly 24 h before SAH constructed, the vehicle groups received normal saline (NS) injection (*n* = 6, each group, Figure 2). OSM (hepcidin inducer) at doses of 100 ng, 1, 5, and 10 *μ*g was injected intracerebroventricularly, the vehicle groups received a phosphate-buffered saline (PBS) injection (*n* = 6, each group, Figure 2). Western blot to evaluate the expression of hepcidin, DMT1, FPN1, and GPX4 to get the proper concentration of ebselen, heparin and OSM. The proper concentration of ebselen, heparin and OSM were chosen to evaluate ferroptosis and EBI after SAH (*n* = 6, each group, Figure 2).

#### 2.3.3. Treatment 3

The proper concentration of OSM and ebselen were injected intracerebroventricularly 24 h before SAH constructed. The vehicle groups received equal volumes of PBS and Abs (*n* = 6, each group, Figure 2).

### 2.4. Immunofluorescence

Rats were sacrificed 24 h after perforation for immunofluorescence staining using the methods described in our previous study. Ten-micrometer sections of brains from rats with SAH were incubated with the following primary antibodies overnight at 4°C: anti-hepcidin (1 : 100, Abcam, ab30760), anti-DMT1 (1 : 300; Bioss, bs-3577R), and anti-NeuN (1 : 100, Millipore, MAB377X). After washing in PBST, sections were incubated with the following fluorescent secondary antibodies at 37°C for 1.5 h: Alexa Fluor 555 (1 : 50, Beyotime, A0453) and Alexa Fluor 488 (1 : 50, Protech, SA00006-1). Fluorescence microscopy (Nikon A1 + R, Japan) was used to detect the protein locations in the neurons.

### 2.5. Western Blotting

Western blotting was performed 24 h after SAH. Equal amounts of total protein (50 *μ* g) were loaded onto a sodium dodecyl sulfate polyacrylamide gel, electrophoresed, and transferred to a polyvinylidene fluoride (PVDF) membrane. The membranes were incubated at 37°C with 5% nonfat milk in Tris-buffered saline that contained 0.1% Tween 20 (TBST) for 2 h, followed by incubation with primary antibodies overnight at 4°C. The following primary antibodies were used: anti-hepcidin (1 : 100, Abcam, ab30760), anti-DMT1 (1 : 300, Bioss, bs-3577R), anti-FPN1 (1 : 1000, Protech, 26601-1-AP), and anti-GPX4 (1 : 1000, Protech, 14432-1-AP). The membranes were incubated with the goat anti-rabbit HRP-conjugated secondary antibody (1 : 2000, BosterBio, BA1054) after washing in TBST. The membranes were visualized and analyzed using Fusion FX7 Spectra chemiluminescence imaging system (Vilber, France).

### 2.6. Perl's Iron Staining

Samples were collected 24 h after SAH. The Perl's iron staining was performed as described previously with some modifications [28]. Paraffin slices 10 microns thick were taken from the brains with SAH rats and incubated with xylene I and xylene II for 15 min, followed by hydration with 100%, 95%, 85%, 80%, 75% and 70% alcohol solutions for 1 min. Incubated with Perl's iron staining (80 ml 20% hydrochloric acid and 80 ml 10% potassium ferrocyanide mixed for 5 min) for 20-30 min and washed with distilled water 3 times. After 1 min of H&E staining, sections were rapidly dehydrated for 5 s at 80%, 85%, 90%, and 100% alcohol concentrations. Finally, the sections were cleaned with xylene and sealed with resin. The stained sections were examined microscopically and multiple fields were evaluated. According to the manufacturer's instructions, Perl's iron staining was introduced into the study to obtain staining with greater detail.

### 2.7. Iron Content

Samples were collected 24 h after SAH and homogenized with physiological saline at a ratio of 1 : 9. The iron content was measured using a rat iron assay kit (Nanjing Jian Cheng, A039-2) according to the manufacturer's instructions.

### 2.8. MDA

Samples were collected 24 h after SAH and LPO was measured based on the MDA content using an MDA assay kit (Nanjing Jian Cheng, A0003-1) according to the manufacturer's instructions.

### 2.9. GSH

Samples were collected 24 h after SAH and GSH was measured using a GSH assay kit (Nanjing Jian Cheng, A006-2) according to the manufacturer's instructions.

### 2.10. GPX4 Activity

Samples were collected 24 h after SAH and GPX4 was measured using a rat GPX4 assay kit (Nanjing Jian Cheng, A039-2) according to the manufacturer's instructions.

### 2.11. Transmission Electron Microscopy (TEM)

The brains of the SAH rats were stored 24 h after perfusion with 4% glutaraldehyde solution, and the cortex of each perfused brain was sliced to a 1∗1∗1 mm size to obtain slices for TEM. Prior to TEM, these slices were subjected to fixing, dehydrating, embedding, curing, thin-slice machine slicing at 50-60 nm, and double staining with 3.3% uranium-citric acid.

### 2.12. Neurological Deficit Scores

The scoring method was introduced to evaluate neurological deficit [26], the following six separate tests were scored 24 h and 72 h after operation to evaluate the prognosis of SAH rats: independent movement, limb movement symmetry, climbing movement, forepaw extension, response to touch stimulation and beard response. The neurological deficit scores were evaluated by an observer who did not know the group ID and treatment, and Dengzhi Jiang and Qing Zhao scored. The lower the scores, the more serious the neurological impairment.

### 2.13. Evans Blue Staining

The permeability of blood brain barrier was evaluated by Evans blue staining 72 h after SAH, as described previously by He et al. [27] with minor modifications. The rats were anesthetized and directly injected with 2% Evans blue solution (8 ml/kg, Beyotime) through femoral vein. Evans blue solution was allowed to circulate for 3 hours before transcardiac perfusion. After transcardiac perfusion with PBS (pH 7.4), the rats were euthanized and the left and right cerebral hemispheres were stored at −80°C before use. Brain tissue was homogenized in 99% dimethylformamide, and the samples were cultured in 50°C water bath for 48 hours. After centrifugation for 30 minutes (12000 g at 4°C), the supernatant was collected and the absorbance of the sample was measured at 620 nm using a spectrophotometer.

### 2.14. Analysis of Brain Water Content

As mentioned in previously reported [27], the brain water content of rats was detected at 72 h after SAH induction. Anesthetized the animals and immediately removed the brain. The brain was divided into four parts: the right hemisphere, the left hemisphere, the cerebellum and the brain stem. Weighed each part on an electronic analytical balance to obtain a wet weight (a) and then dried at 100°C for 24 h to obtain the dry weight (b)). The brain water content (c) was calculated by the following formula: c = (a-b)/a.

### 2.15. Statistical Analysis

The data are expressed as the Means ± SEM. Statistical differences among the groups were analyzed using one-way ANOVA followed by Tukey's post hoc test. Mortality was evaluated using Fisher's exact test. A p value of <0.05 was considered statistically significant. All statistical analyses were performed using GraphPad Prism 5 for Windows (GraphPad software, San Diego, California).

## 3. Results

### 3.1. Hepcidin and DMT1 Were Upregulated following the Time Course in EBI after SAH

**Figure 3:**
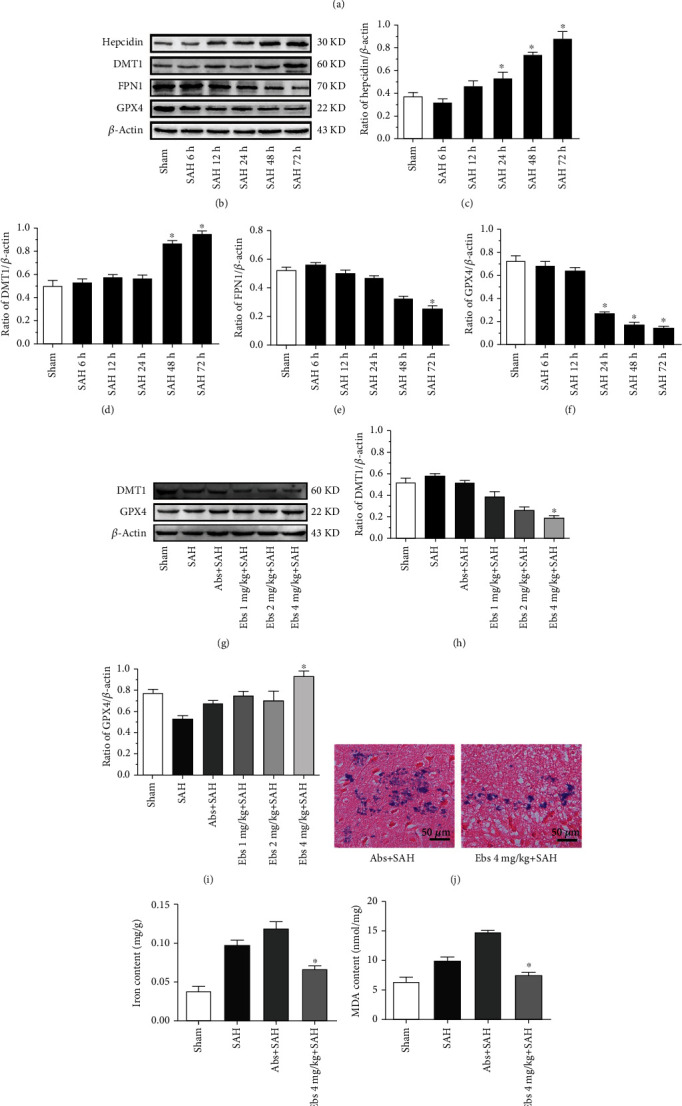
Immunofluorescence staining for hepcidin, DMT1 and GPX4 showed co-localization in neurons 24 h after SAH. Hepcidin, DMT1 and GPX4 were co-localized with NeuN-positive cells (neurons); hepcidin (red), DMT1 (red), GPX4 (red) NeuN (green), DAPI (nucleus, blue); original magnification 400× (a) (*n* = 6). Expression of hepcidin, DMT1, FPN1 and GPX4 at different time points after SAH. Representative WBs of hepcidin, DMT1, FPN1 and GPX4 (b). Densitometric quantification of the optical densities of these protein bands (c-f). All protein expression levels were significantly changed at 24-72 h after SAH, ∗*P* <0.05 vs the sham group (*n* = 6, each group). Expression of DMT1 and GPX4 after different doses of ebselen were introduced following SAH. Representative WBs of DMT1 and GPX4 (G), densitometric quantification of the optical densities of these protein bands (h-i), all protein expression levels were significantly changed at the dose of 4 mg/kg ebselen after SAH, ∗*P* <0.05 vs the Abs+SAH group, (*n* = 6, each group). Perl's staining of iron after 4 mg/kg ebselen was administered. Positive areas 4 mg/kg ebselen decreased, compared with the Abs+SAH groups, final magnification 400× (j) (*n* = 6, each group). The activity of components of ferroptosis (iron content, MDA, GSH, and GPX4) were determined after 4 mg/kg ebselen was introduced. Treatment with 4 mg/kg ebselen decreased MDA while increasing GSH and GPX4 activity and protecting against ferroptosis, compared with the Abs+SAH groups (k-n), ∗*P* <0.05 vs the Abs+SAH group (*n* = 6, each group).

Double immunofluorescence staining revealed that hepcidin, DMT1, and GPX4 were mainly present in the cytoplasm of cortical neurons after SAH (*n* = 6, Figure 3(a)). Western blot analysis was performed to determine the expression profiles of hepcidin, DMT1, FPN1, and GPX4. at 6, 12, 24, 48, and 72 h after SAH (Figure 3(b)). The Western blot results indicated that the hepcidin and DMT1 levels increased with time, and in particular hepcidin was significantly increased from 24 h to 72 h (p <0.05) (*n* = 6, Figure 3(c)). DMT1 was increased at 6 and 12 h, decreased at 24 h, and significantly increased from 48 h to 72 h (p <0.05) (*n* = 6, Figure 3(d)). In contrast, FPN1 was decreased at 6 h, and significantly decreased at 72 h (p <0.05) (*n* = 6, Figure 3(e)). GPX4 gradually decreased during the first 72 h, significantly decreased from 24 h to 72 h (p <0.05) (*n* = 6, Figure 3(f)), compared with the sham group.

### 3.2. DMT1 Inhibition Influenced Iron Metabolism, Resulting in Diminishing Iron Content and Cellular Distribution of Iron Deposits, and Protected against Ferroptosis in EBI following SAH

A suppressant of DMT1, ebselen, was intracerebroventricularly injected at three doses (1, 2, and 4 mg/kg) 24 h before SAH. The expression of DMT1 was significantly decreased with 4 mg/kg ebselen (p <0.05), whereas 1 and 2 mg/kg ebselen had no effect on DMT1 (p >0.05) (*n* = 6, Figure 3(h)), compared with the Abs+SAH. GPX4 was significantly increased with 4 mg/kg ebselen (p <0.05), whereas 1 and 2 mg/kg ebselen had no influence on GPX4 (p >0.05) (*n* = 6, Figure 3(i)), compared with the Abs+SAH. Ebselen (4 mg/kg) had a significant effect on DMT1 and GPX4.

**Figure 4:**
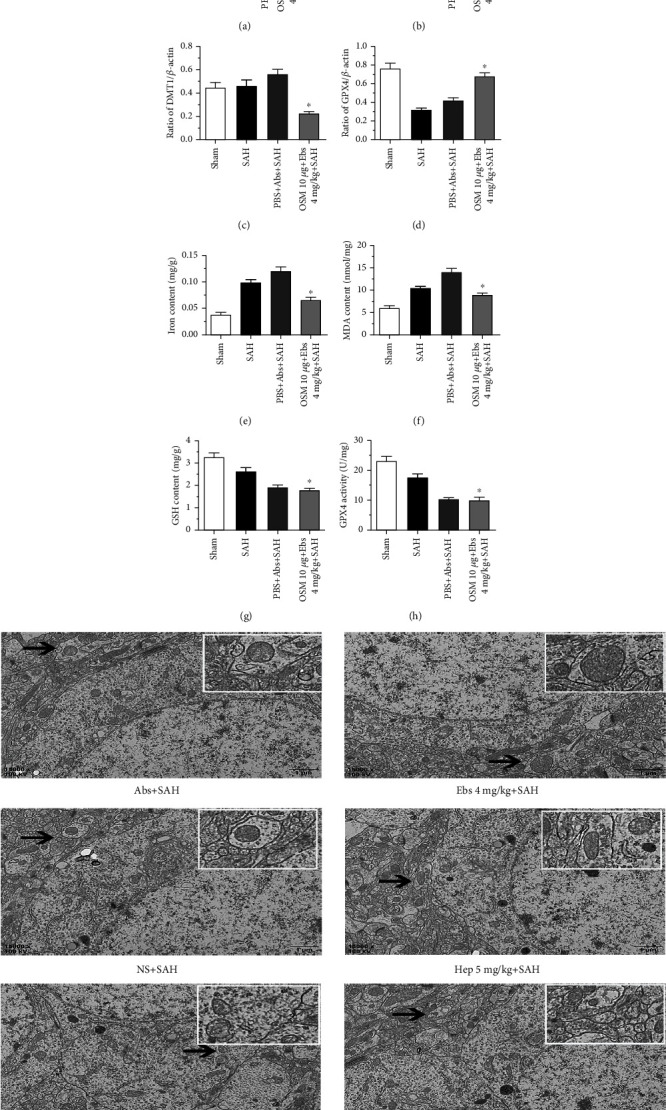
Both OSM and ebselen were used, the expression of hepcidin was improved (b), DMT1 was reduced (c), while GPX4 was increased (d),∗*P* <0.05 vs PBS + SAH group (*n* = 6, each group). 4 mg/kg ebselen could reverse the effects of 10 *μ*g OSM on iron metabolism and ferroptosis (e–h), ∗*P* <0.05 vs PBS + SAH group (*n* = 6, each group). 5 mg/kg heparin protected against ferroptosis, which reduced size of mitochondria in TEM after SAH, compared to the NS + SAH; 4 mg/kg ebselen decreased the size of mitochondria of ferroptosis after SAH, and it protected against ferroptosis, compared to the Abs+SAH; OSM 10 *μ*g aggravated ferroptosis, which augmented the size of mitochondria in TEM after SAH, compared to the PBS + SAH (i) (*n* = 6, each group).

**Figure 5:**
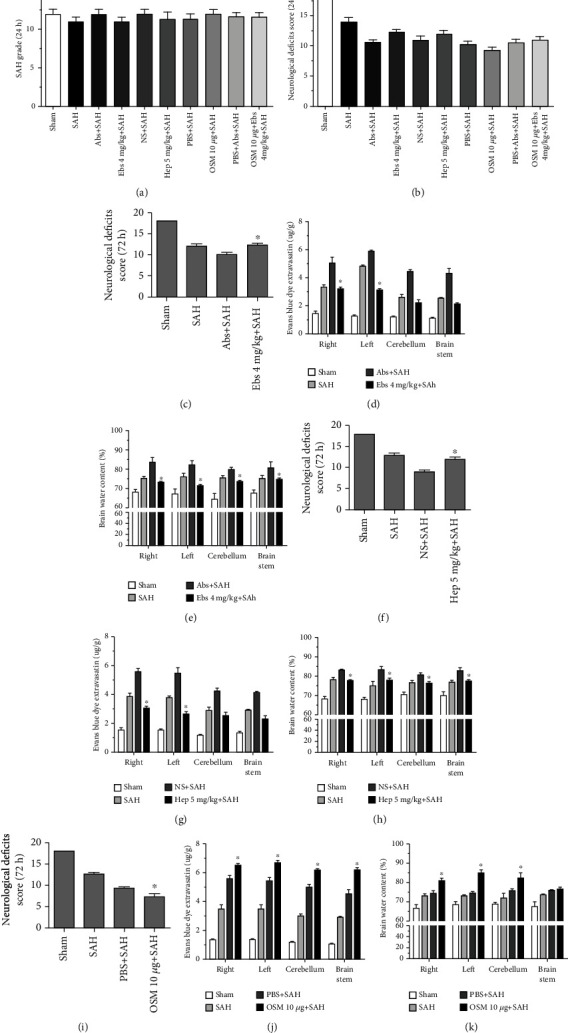
The SAH grading scores were similar among each surgery group (*n* = 6, Fig.  4(a), p =0.852), The neurological deficit scores among each surgery group (*n* = 6, Fig.  4(b), p =0.806) along with the evaluation of SAH grade. Treatment with 4 mg/kg ebselen to determine the EBI (c-e), 4 mg/kg ebselen decreased neurological function deficit compared with the SAH and Abs+SAH groups (c). BBB permeability was also alleviated in four areas (LH, RH, CB, and BS) after treatment with 4 mg/kg ebselen (d). Brain water content was decreased in four areas (LH, RH, CB, and BS) after treatment with 4 mg/kg ebselen (e), ∗*P* <0.05 vs Abs+SAH group (*n* = 6, each group). Treatment with 5 mg/kg heparin to determine the EBI (f-h), 5 mg/kg heparin decreased neurological functions (f). BBB permeability based on Evans blue staining in the LH and RH was decreased by the 5 mg/kg heparin treatment (g). Brain water content was decreased in four areas (LH, RH, CB, and BS) after treatment with 5 mg/kg heparin (h), ∗*P* <0.05 vs NS + SAH group (*n* = 6, each group). Treatment with 10 *μ*g OSM to determine the EBI (i-k), 10 *μ*g OSM aggravated neurological functions (i). BBB permeability based on Evans blue staining in the LH and RH was increased by the 10 *μ*g OSM treatment (j). Brain water content was increased in four areas (LH, RH, CB, and BS) after treatment with 10 *μ*g OSM (k), ∗*P* <0.05 vs PBS + SAH group (*n* = 6, each group).

Based on the Western blot results, the appropriate dose of ebselen (4 mg/kg) was administered to examine the role of DMT1 signaling in EBI, iron metabolism, and ferroptosis. Ebselen at a dose of 4 mg/kg could influence iron metabolism, resulting in decreases in the Perl's iron staining and iron content (Figures 3(j), 3(k)). Ferroptosis was evaluated basing on LPO production of MDA, and GSH content and GPX4 activity were decreased (p <0.05) (*n* = 6, Figures 3(l), 3(m), 3(n)), compared with the Abs+SAH groups. Additionally, 4 mg/kg ebselen decreased the size of mitochondria of ferroptosis after SAH, and it protected against ferroptosis (*n* = 6, Figure 4(i)). The results indicated that 4 mg/kg ebselen improved the neurological deficit, ameliorated the brain edema, and decreased the blood-brain barrier permeability, thus mitigating EBI (*n* = 6, Figures 5(c), 5(d), 5(e)).

### 3.3. Hepcidin Suppression Decreased the Iron Content and Perl's Iron Staining and Conferred Protection against Ferroptosis and EBI after SAH by Inhibiting DMT1

**Figure 6:**
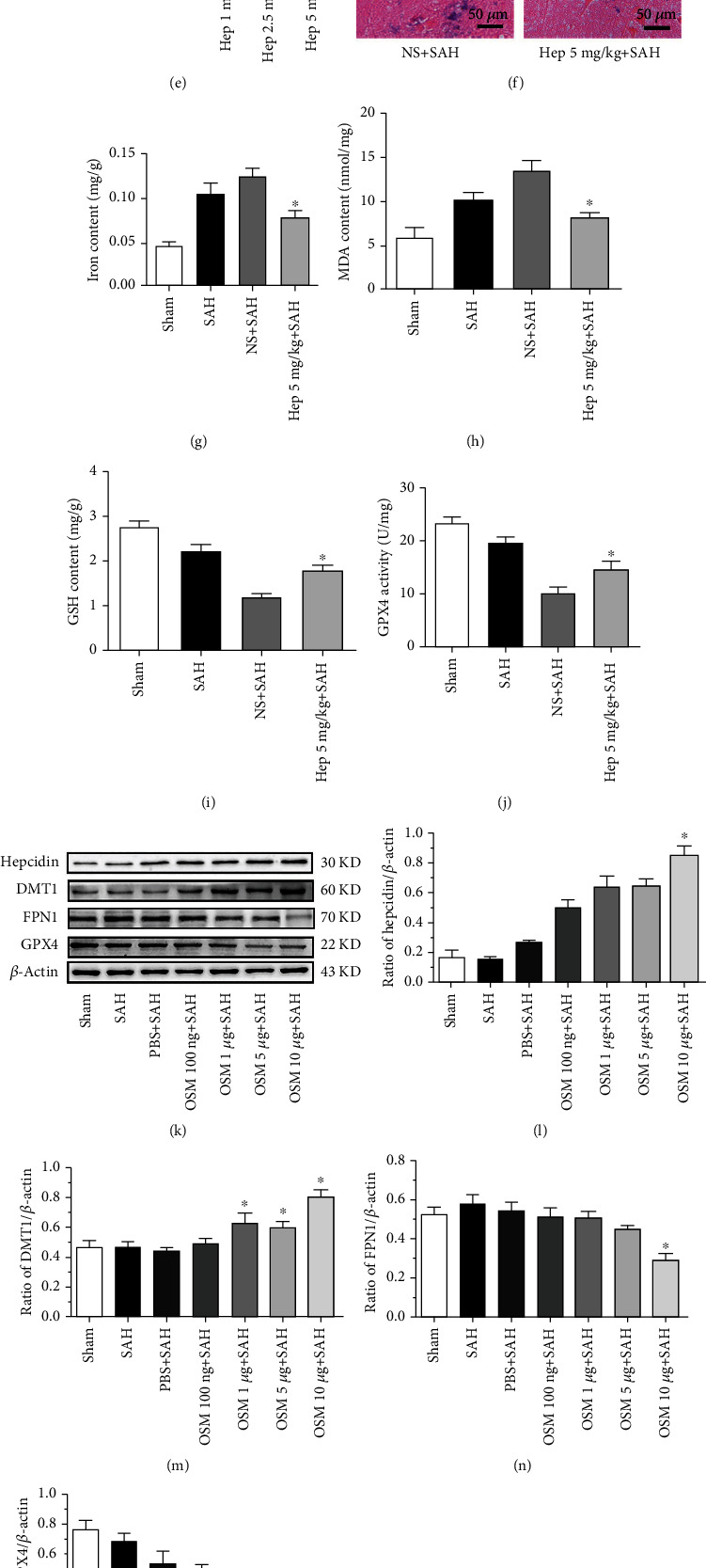
Expression of hepcidin, DMT1, FPN1 and GPX4 after different doses of heparin were introduced following SAH. Representative WBs of hepcidin, DMT1, FPN1 and GPX4 (a), densitometric quantification of the optical densities of these protein bands (b-e), all protein expression levels were significantly changed at the dose of 5 mg/kg heparin after SAH, ^∗^*P* <0.05 vs the NS + SAH group (*n* = 6, each group). Perl's iron staining after 5 mg/kg heparin was administered. Positive areas 5 mg/kg heparin decreased, compared with the NS + SAH groups, final magnification 400× (f). The activity of components of ferroptosis (iron content, MDA, GSH, and GPX4) were determined after 5 mg/kg heparin was introduced. Treatment with 5 mg/kg heparin decreased iron content and MDA, while increasing GSH and GPX4 activity and protecting against ferroptosis, compared with the NS + SAH groups (g-j), ^∗^*P* <0.05 vs the NS + SAH group (*n* = 6, each group). Expression of hepcidin, DMT1, FPN1, and GPX4 after different doses of OSM were introduced following SAH. Representative WBs of hepcidin, DMT1, FPN1, and GPX4 (k), densitometric quantification of the optical densities of these protein bands (l-o), and all protein expression levels were significantly changed at the dose of 10 *μ*g OSM after SAH, ^∗^*P* <0.05 vs. the NS+SAH group (*n* = 6, each group). Perl's iron staining after 10 *μ*g OSM was administered. Positive areas 10 *μ*g OSM increased, compared with the PBS+SAH groups, final magnification ×400 (p). The activity of components of ferroptosis (iron content, MDA, GSH, and GPX4) were determined after 10 *μ*g OSM was introduced. Treatment with 10 *μ*g OSM increased iron content and MDA, while decreasing GSH and GPX4 activity and aggravating ferroptosis, compared with the PBS+SAH groups (q-t), ^∗^*P* <0.05 vs. the PBS+SAH group (*n* = 6, each group).

A hepcidin antagonist, heparin, was administered (1, 2.5, and 5 mg/kg) via intracerebroventricular injection 24 h before SAH induction. The Western blot results indicated that hepcidin and DMT1 both decreased, whereas FPN1 and GPX4 increased after heparin injection. Heparin (1, 2.5 and 5 mg/kg) decreased the expression of hepcidin, and the effect of 5 mg/kg heparin was most robust (p <0.05) (*n* = 6, Figure 6(b)), compared with the NS + SAH. DMT1 expression was significantly decreased with heparin at doses of 2.5 mg/kg and 5 mg/kg (p <0.05) (*n* = 6, Figure 6(c)), compared with the NS + SAH. FPN1 expression was significantly increased with heparin at doses of 5 mg/kg (p <0.05) (*n* = 6, Figure 6(d)), compared with the NS + SAH. GPX4 was significantly increased with heparin at a dose of 5 mg/kg (p <0.05) (*n* = 6, Figure 6(e)), compared with the NS + SAH groups. Heparin at 5 mg/kg had a significant effect on the expression of hepcidin, DMT1, FPN1 and GPX4 (*n* = 6, Figure 6(a)).

Out of heparin doses, 5 mg/kg heparin was administered to evaluate iron metabolism, ferroptosis and EBI. The results indicated that 5 mg/kg heparin decreased the Perl's iron staining and iron content (p <0.05) (Figures 6(f), 6(g)), compared with the NS + SAH groups. It also decreased the MDA content and increased the GSH content and GPX4 activity (p <0.05) (*n* = 6, Figures 6(h), 6(i), 6(j)), compared with the NS + SAH groups. Additionally, 5 mg/kg heparin protected against ferroptosis, which reduced the size of mitochondria in TEM after SAH (*n* = 6, Figure 4(i)). It also improved the neurological deficits, reduced the brain edema, and decreased the blood-brain barrier permeability, thus mitigating EBI (p <0.05) (*n* = 6, Figures 5(f), 5(g), 5(h)), compared with the NS + SAH groups.

### 3.4. Hepcidin Stimulation Promoted the Expression of DMT1, Influenced Iron Metabolism, and Aggravated EBI and Ferroptosis after SAH

An inducer of hepcidin, OSM, was used to verify the role of hepcidin in SAH-induced brain injury. OSM was administered (100 ng, 1 *μ*g, 5 *μ*g, and 10 *μ*g per rat) 24 h before SAH. The Western blot results indicated that OSM enhanced the expression of hepcidin and DMT1, whereas GPX4 expression was reduced. OSM induced the expression of hepcidin, while 10 *μ*g OSM even more potently stimulated the expression of hepcidin (p <0.05) (*n* = 6, Figure 6(l)), in comparison with the PBS + SAH groups. OSM at doses of 1 *μ*g, 5 *μ*g and 10 *μ*g significantly stimulated the expression of DMT1 (p <0.05) (*n* = 6, Figure 6(m)), as compared to the PBS + SAH groups. OSM at doses of 10 *μ*g significantly inhibited the expression of FPN1 (p <0.05) (*n* = 6, Figure 6(n)), as compared to the PBS + SAH groups. OSM at doses of 100 ng, 1 *μ*g, 5 *μ*g, and 10 *μ*g per rat decreased the expression of GPX4, whereas 10 *μ*g OSM very potently alleviated the expression of GPX4 (p <0.05) (*n* = 6, Figure 6(o)), in contrast to the PBS + SAH groups. Thus, OSM at a dose of 10 *μ*g had a very significant effect on the expression of hepcidin, FPN1, DMT1 and GPX4 as well (*n* = 6, Figure 6(k)).

Therefore, OSM at a dose of 10 *μ*g per rat was employed to detect the components of ferroptosis. OSM at 10 *μ*g promoted iron metabolism and ferroptosis, by increasing and Perl's iron staining and the iron content (p <0.05) (*n* = 6, Figures 6(p), 6(q)), in comparison with the PBS + SAH groups. It also increased the MDA content, decreased the GSH content and GPX4 activity (p <0.05) (*n* = 6, Figures 6(r), 6(s), 6(t)), in comparison with the PBS + SAH. Additionally, OSM 10 *μ*g aggravated ferroptosis, which augmented the size of mitochondria in TEM after SAH (*n* = 6, Figure 5(i)), in comparison with the PBS + SAH. 10 *μ*g OSM also decreased the neurological deficits, increased the brain edema, and increased the blood-brain barrier permeability, thus aggravated EBI (p <0.05) (*n* = 6, Figure 6(i), 6(j), 6(k)), compared with the NS + SAH groups.

### 3.5. Induction of Hepcidin Promoted Iron Metabolism Enhancement and Ferroptosis, Was Reversed via DMT1 Inhibition in SAH Rats

The effects of OSM and ebselen on hepcidin and DMT1 were revealed as above,10 *μ*g OSM and 4 mg/kg ebselen were both employed to validate the effects of hepcidin in SAH (*n* = 6, Figure 4(a)). With this combined treatment, Western blot results indicated that the expression of hepcidin was improved (p <0.05) (*n* = 6, Figure 4(b)). Moreover, DMT1 was reduced while GPX4 was increased (p <0.05) (*n* = 6, Figures 4(c), 4(d)), as compared with the PBS + Abs+SAH groups. According to the results presented above, 10 *μ*g OSM elevated the iron content, when it was used alone in SAH. Whereas 4 mg/kg ebselen could reverse the effects of 10 *μ*g on iron content, when both OSM and ebselen were used, compared with the PBS + Abs+SAH groups. Therefore, 4 mg/kg ebselen could reverse the effects of 10 *μ*g OSM on iron metabolism and ferroptosis (*n* = 6, Figures 4(e), 4(f), 4(g), 4(h)).

### 3.6. SAH Severity and Mortality

SAH grade and mortality are prognostic indicators, which recorded after surgery and treatment. The SAH grading scores were similar among each surgery group (*n* = 6 Fig.  5(a), p =0.852), The neurological deficit scores among each surgery group (*n* = 6, Fig.  5(b), p =0.806), along with the evaluation of SAH grade. A total of 30 rats died during or after model constructed due to severe SAH, and another 21 rats were excluded because of their low SAH grade. No death was observed in the sham group, after surgery, with mortality rates (Table 1), which were calculated as follows: sham = 0% (0 of 40), SAH = 27.27% (18 of 66), NS = 25.0% (6 of 24), Hep = 16.67% (6 of 36), Abs = 17.39% (4 of 23), Ebs = 17.65% (9 of 51), PBS = 26.92% (7 of 26), OSM = 21.28% (10 of 47) and PBS + Abs = 25% (4 of 16), OSM + Ebs = 29.17% (7 of 24). However, there were no statistically significant differences in mortality rates between treatment groups after analysis with a Fisher two-sided exact test.

## 4. Discussion

EBI after SAH remains a major cause of high lethality and disability of patients, and previous studies have verified that iron metabolism disturbances play an important role in SAH [29]. Ferroptotic cell death is morphologically, biochemically and genetically distinct from apoptosis, various forms of necrosis, and autophagy [30, 31] The process of ferroptosis is characterized by the overwhelming, iron-dependent lethal accumulation of lipid ROS, and unlike other forms of apoptotic and nonapoptotic death, this requirement for ROS accumulation appears to be indispensable in ferroptosis [32]. Dixon et al. first explored the mechanism of ferroptosis, which is dependent on intracellular iron and the accumulation of LPO [5]. The main observed morphological effect was a decrease in the size of mitochondria, whereas the iron content and LPO products, also known as lipid ROS, increased to induce ferroptosis, accompanied by a decreased GSH content and GPX4 activity [9, 33]. Exogenous iron could boost the components of ferroptosis and morphological aspects of cell death, as previously described, and it is believed that disturbed iron metabolism is primarily responsible for ferroptosis [34, 35]. The cerebral occurrence of ferroptosis has been observed in Parkinson's disease, and may occur in several types of brain injury [36], as well as in infection [37] and cancer [38, 39]. Ferroptosis has been found occur in EBI after SAH, lines reported that via reduction of lipid peroxidation and inhibiting p53 could alleviate ferroptosis in EBI after SAH [40]. Notably, ferroptosis strongly relates to iron metabolism but whether influencing iron metabolism could alleviate ferroptosis and the pathway have not been reported.

Evidence has indicated that the only intracellular iron transporter, DMT1, may mediate intracellular iron metabolism [41], and be involved in ferroptosis induction, while inhibiting DMT1 by ebselen may modulate the iron uptake [18]. Moreover, hepcidin is a major iron metabolism hormone and may influence the iron content in the ischemic brain, and lines reported that hepcidin could increase DMT1 and inhibit FPN1 expression in regulating iron metabolism [6, 42]. Recently, several lines of evidence have shown that recombinant hepcidin could stimulate the expression of DMT1, and suppressing hepcidin may decrease brain iron content [43, 44]. Thus, we hypothesized that hepcidin might have an effect on iron metabolism and ferroptosis, via DMT1 activation, in EBI after SAH (Figure 1).

To validate the effect of hepcidin in SAH, we examined the expression of hepcidin and DMT1, FPN1 over time in EBI after SAH, and the results showed that both hepcidin and DMT1 increased, FPN1 decreased. Whereas GPX4, which serves as the main regulator of ferroptosis, decreased compared with the sham group. These findings are consistent with our previous study, which demonstrated that SAH stimulates the expression of hepcidin. Yang et al. found that cancer cell death occurred in the form of ferroptosis, in which GPX4 decreased [42]. In our previous investigation, we also determined that the expression of GPX4, the protein protective against ferroptosis decreased in SAH. These findings prompted our hypothesis that SAH might cause cell death in the form of ferroptosis.

In this study, we obtained several fruitful results as follows. Firstly, the iron metabolism related proteins, hepcidin and DMT1 were upregulated, while, FPN1 decreased, at the same time, the GPX4 also decreased in EBI after SAH. Secondly, the only intracellular iron transporter, DMT1, has been suggested to induce intracellular iron release and lead to iron deposition, at last induced ferroptosis. An inhibitor of DMT1, ebselen, was employed to verify the effects of DMT1 on iron metabolism and ferroptosis following SAH. The findings revealed that ebselen could decrease the iron content and Perl's iron staining and alleviate ferroptosis in SAH. Thirdly, an inhibitor and an inducer of hepcidin were administered to detect the effects of hepcidin on iron metabolism and ferroptosis in EBI after SAH. The findings proved that the inhibitor of hepcidin, heparin, could decrease DMT1, increase FPN1, also alleviate the iron content, Perl's iron staining, and ferroptosis in SAH, consistent with the effect of ebselen on DMT1; moreover, heparin could also protect against EBI. The results suggested that the inducer of hepcidin, OSM, could increase DMT1, decrease FPN1, also elevate the iron content, enhance Perl's iron staining, and aggravate ferroptosis and EBI. Finally, the results suggested that hepcidin could influence iron metabolism through DMT1, induce iron accumulation, promote LPO accumulation, and then lead to ferroptosis. The inducer of hepcidin and the inhibitor of DMT1, OSM and ebselen, respectively, were both used to investigate the effects of hepcidin on metabolism and ferroptosis via DMT1 in EBI after SAH. The results suggested that ebselen could reverse the effects of OSM and hepcidin on metabolism and ferroptosis.

However, several shortcomings of this study need to be discussed. Different doses of OSM, heparin and ebselen were administered in this research, however, the safety of these doses of drugs remains to be verified. Although gradient concentrations of OSM, heparin and ebselen were introduced in this research, only the most significant influences of the doses of OSM, heparin and ebselen on various protein levels were adopted to investigate ferroptosis and EBI. Other doses of OSM, heparin and ebselen need to be investigated to confirm that OSM, heparin and ebselen have conclusive impacts on ferroptosis and EBI after SAH. However, it needs to be stressed that this study still examined many doses dependent effects, which is not always the case regarding other SAH studies. The results indicated that when doses of OSM and ebselen were both introduced at the same time, they had an influence on hepcidin, DMT1, and GPX4 but did not have significant effects on ferroptosis or EBI. This may indeed indicate that hepcidin-induced aggravation of EBI after SAH requires DMT1 involvement. Lastly, heparin is an anticoagulant drug, to avoid its anticoagulant we introduced the glycol-split non-anticoagulant heparin which lost antithrombin-binding affinity, the anticoagulant of glycol-split non-anticoagulant heparin was studied in HepG2 cells is effective, but whether the anticoagulant of the applied dose of glycol-split non-anticoagulant is effective in SAH, whether it could aggravate bleeding in SAH needs further investigation.

The results of this present study strongly point towards DMT1 as a significant mediator of ferroptosis in the hemorrhagic brain. Both hepcidin and DMT1 appear to be involved in lipid peroxidation and GSH depletion after SAH, which together with a reduced GPX4 activity and increased iron content may lead to EBI via aggravated ferroptosis in several cell compartments.

Concordantly, the suppression of hepcidin and DMT1 alleviates ferroptosis and EBI after SAH. In contrast, the activation of hepcidin aggravates these SAH-induced alterations, and this effect is DMT1-dependent. Although further studies are needed, present results provide a novel insight into the disturbed iron homeostasis that may play a central role in mechanisms of EBI after SAH.

## 5. Conclusions

Inhibition of DMT1 by ebselen could suppress iron accumulation and lipid peroxidation, and thereby alleviate ferroptosis and EBI in SAH rats. Heparin downregulated the expression of hepcidin and DMT1, and exerted protective effects that were equivalent to those of ebselen. In addition, OSM increased the expression of hepcidin and DMT1 and aggravated ferroptosis and EBI, while it was reversed by ebselen. Therefore, the study revealed that hepcidin could regulate iron metabolism and contribute to ferroptosis via DMT1 signaling activation in rats with EBI after SAH.

## Figures and Tables

**Table 1:** SAH grade and mortality are prognostic indicators, which recorded after surgery and treatment. A total of 30 rats died during or after model constructed due to severe SAH, and another 21 rats were excluded because of their low SAH grade. No death was observed in the sham group, after surgery, with mortality rates (Table 1), which were calculated as follows: sham = 0% (0 of 40), SAH = 27.27% (18 of 66), NS = 25.0% (6 of 24), Hep = 16.67% (6 of 36), Abs = 17.39% (4 of 23), Ebs = 17.65% (9 of 51), PBS = 26.92% (7 of 26), OSM = 21.28% (10 of 47) and PBS + Abs = 25% (4 of 16), OSM + Ebs = 29.17% (7 of 24). However, there were no statistically significant differences in mortality rates between treatment groups.

Groups	Mortality	Excluded	Groups	Mortality	Excluded
*Experiment 1*			*Experiment 2*		
Sham	0%(0/8)	0	Sham	0% (0/8)	0
SAH (6 h 12 h, 24 h, 48 h, 72 h)	21.8% (7/32)	3	SAH	37.5% (3/8)	1
*Experiment 3*			SAH + Abs	20% (2/10)	0
Sham	0% (0/8)	0	SAH + Ebs (1 mg/kg)	16.67% (2/12)	1
SAH	37.5%(3/8)	1	SAH + Ebs (2 mg/kg)	23.07 (3/13)	1
SAH + NS	20% (2/10)	0	SAH + Ebs (4 mg/kg)	20% (3/15)	0
SAH + Hep (1 mg/kg)	12.5% (1/8)	1	*Experiment 5*		
SAH + Hep (2.5 mg/kg)	22.22 (2/9)	1	Sham	—	0
SAH + Hep (5 mg/kg)	11.1% (1/9)	0	SAH	—	1
*Experiment 4*			SAH + PBS + Abs	25% (4/16)	1
Sham	0% (0/8)	0	SAH + OSM (10 *μ*g) + Ebs (4 mg/kg)	29.1% (7/24)	
SAH	25% (2/8)	2	*Experiment 6*		0
SAH + PBS	27.78% (5/18)	0	Sham	0% (0/8)	0
SAH + OSM (100 ng)	33.33% (3/9)	0	SAH	30% (3/10)	1
SAH + OSM(1 *μ*g)	20% (2/10)	1	SAH + NS	28.6% (4/14)	0
SAH + OSM (5 *μ*g)	20% (2/10)	1	SAH + Hep (5 mg/kg)	20% (2/10)	0
SAH + OSM(10 *μ*g)	10% (1/10)	1	SAH + Abs	15.38% (2/13)	1
			SAH + Ebs (4 mg/kg)	9.1% (1/11)	0
			SAH + PBS	20% (2/8)	2
			SAH + OSM (10 *μ*g)	25% (2/8)	1
Total		11	Total		10

## Data Availability

All the data supporting the results were shown in the paper and can be available from the corresponding authors.
